# A simulation analysis to characterize the dynamics of vaccinating behaviour on contact networks

**DOI:** 10.1186/1471-2334-9-77

**Published:** 2009-05-28

**Authors:** Ana Perisic, Chris T Bauch

**Affiliations:** 1 Department of Mathematics and Statistics, University of Guelph, Guelph, Ontario, N1G 2W1, Canada

## Abstract

**Background:**

Human behavior influences infectious disease transmission, and numerous "prevalence-behavior" models have analyzed this interplay. These previous analyses assumed homogeneously mixing populations without spatial or social structure. However, spatial and social heterogeneity are known to significantly impact transmission dynamics and are particularly relevant for certain diseases. Previous work has demonstrated that social contact structure can change the individual incentive to vaccinate, thus enabling eradication of a disease under a voluntary vaccination policy when the corresponding homogeneous mixing model predicts that eradication is impossible due to free rider effects. Here, we extend this work and characterize the range of possible behavior-prevalence dynamics on a network.

**Methods:**

We simulate transmission of a vaccine-prevetable infection through a random, static contact network. Individuals choose whether or not to vaccinate on any given day according to perceived risks of vaccination and infection.

**Results:**

We find three possible outcomes for behavior-prevalence dynamics on this type of network: small final number vaccinated and final epidemic size (due to rapid control through voluntary ring vaccination); large final number vaccinated and significant final epidemic size (due to imperfect voluntary ring vaccination), and little or no vaccination and large final epidemic size (corresponding to little or no voluntary ring vaccination). We also show that the social contact structure enables eradication under a broad range of assumptions, except when vaccine risk is sufficiently high, the disease risk is sufficiently low, or individuals vaccinate too late for the vaccine to be effective.

**Conclusion:**

For populations where infection can spread only through social contact network, relatively small differences in parameter values relating to perceived risk or vaccination behavior at the individual level can translate into large differences in population-level outcomes such as final size and final number vaccinated. The qualitative outcome of rational, self interested behaviour under a voluntary vaccination policy can vary substantially depending on interactions between social contact structure, perceived vaccine and disease risks, and the way that individual vaccination decision-making is modelled.

## Background

Historically, infectious diseases have been a great threat to human security, causing enormous morbidity and mortality [1]. In the 20th century approximately 2.3 million people died on average in wars and conflicts per year [2], but currently infectious diseases are killing more than seven times that number of people annually [3]. For this reason, through use of mathematical models, researchers have been trying to better understand the transmission and control of infectious diseases. Mathematical modeling dates back to the 18th century when, in order to study the effectiveness of variolation, Daniel Bernoulli formulated a model for smallpox in 1760 [4]. Due perhaps to a lack of understanding of the mechanisms of the spread of infectious diseases, the pace of development of mathematical epidemiology slowed down until the beginning of the 20th century [5-7] though it started growing drastically in the middle of the same century [5,7].

Models of disease dynamics often implicitly assume that human behaviour does not change or have an impact on disease transmission. However, individuals may in fact change their behavior during an outbreak according to the changes in their perceived risk of being infected, and their decisions will in turn have consequences at the population level [5]. Incorporating behavior into models of disease transmission is therefore very important in some cases since it can significantly influence infection dynamics. Consider, for instance, the effect of introducing a voluntary vaccine on infection dynamics: individuals' decision to vaccinate or not likely depends on their perceptions of risk from the vaccine and the infection. Vaccination protects not only those who are vaccinated but also others in the population who are, due to herd immunity, less likely to be infected. Hence, the level of population immunity and therefore the size of an outbreak is collectively determined by individual decisions [8]. As disease prevalence rises, more people will choose to vaccinate. On the other hand, as disease prevalence goes down, people will not favor vaccination allowing the susceptible number of individuals in the population to increase until the disease starts to spread again. Analysis of such behavior-infection models have demonstrated in many cases that if individuals act in their own self interest, eradication of a vaccine preventable disease through voluntary vaccination without economic incentives is difficult or impossible [9-12]. This effect has been variously described in terms of classic game theoretical paradigms such as the "Free Rider Problem", the "Tragedy of the Commons" and the "Prisoner's Dilemma". Game theory has also been applied to vaccination and those studies generally came to similar conclusions [8,13-18]. However, smallpox is the first and only vaccine preventable disease to be globally eradicated under voluntary vaccination policies in many jurisdictions, and without any economic incentives to vaccinate [19]. Considering the predictions of the previous models, how was this possible?

Models that assume a homogeneously mixing population have predictive value for many diseases [20] and have allowed researchers to study many characteristics of an epidemic, such as the existence of threshold values for the spread of an infection [21] and the asymptotic solution for the density of infected people [6,22]. Homogeneous mixing models imply that a susceptible person is equally likely to acquire infection from any infectious person in the population [6,7,23]. This assumption simplifies analysis and is a good approximation for highly transmissible diseases, such as those that spread through aerosol droplets. However, epidemics of close contact infections occur within populations made up of individuals who mostly spend time with close associates and do not mix with other individuals in the population completely at random. Therefore, the homogeneous mixing assumption is not very realistic for diseases that are spread through close contact.

Many infectious diseases, including close contact infections, can be modeled using network models, in which individuals are represented as nodes and contacts between individuals are edges connecting the nodes [24-28]. Contacts between individuals through which a disease spreads, are formed and destroyed according to a set of previously defined rules. The number of edges attached to a node is called the node degree and probability distribution of these degrees over the whole network is called degree distribution. When implemented as agent-based simulations, network models can include any level of detail about the individuals and their relationships in the network. However, researchers often work with mathematical approximations of the contact networks to simplify analysis.

Percolation theory [29,30] and pair approximations [26,31,32] are among many such methods developed in recent years to make predictions of the spread of disease in heterogeneous populations. Results from the previous studies suggest that the spread of an infection can depend significantly on the network structure [29,30,33-36]. Using a number of different techniques such as contact tracking [37], surveys [38], census [30,39,40] and others [36], researchers have been trying to build realistic contact networks. Random networks with regular, Poisson, exponential and scale-free degree distributions have mainly been used in the individual-based models [33,41]. Through use of detailed stochastic network models we have not only been able to understand the relationship between network structure and spread of sexually-transmitted infections [27,28,42], but also how to contain possible bioterrorist attacks [43].

Similarly, it has been found that there are also significant differences in predictions of spatially-structured populations and spatially-unstructured populations [35,44,45]. In spatially-structured populations, the development of coexistence, diversity and altruism is easier [31,46], epidemics have more realistic time series and critical community sizes [32] and evolutionary velocities are slower [47], as compared to spatially-unstructured populations. Researchers have also found that cooperative behavior in classical games such as the Prisoner's Dilemma persists longer in spatially-structured populations [48].

Incorporating human behavior into mathematical models of disease spread is critical for making quantitative predictions about infection dynamics and developing appropriate policies for cases where individual behavior influences disease incidence. At the same time, social contact structure greatly influences infection dynamics and more accurately describes certain diseases. However, most previous models of infectious disease transmission that incorporate human behavior rely upon homogeneous mixing assumptions to a greater or lesser extent, while most spatial or network infectious disease transmission models treat human behavior as fixed.

Earlier research by the authors demonstrated qualitative changes in behavior-infection dynamics once social contact structure is introduced: self-interested behavior can quickly stop outbreaks in discrete, contact-structured populations through voluntary ring vaccination (targeted immunization of primary and secondary contacts) contrary to the predictions of the contact-unstructured populations where self-interested behavior leads to a "tragedy of the commons" [49]. It also illustrated the importance of discrete effects in spatially localized population by showing that events at small, individual scales can have significant implications for population-level outcomes such as final size and number vaccinated. However this model made simplifying assumptions regarding disease natural history, pre-existing immunity, and human behaviour. Our objective in the present paper is to see how relaxing some of these assumptions will affect disease dynamics in a population on a contact-structured network, where individuals choose whether or not to vaccinate against a vaccine-preventable disease by weighing infection risks versus vaccine risks. We also seek to catalog the possible outcomes of behavior-infection dynamics for vaccine-preventable infections on random, static networks. Because these types of models are at an early stage of development, we analyze a simple theoretical model here, in order to identify fundamental types of dynamics that can occur, rather than developing a realistic, highly detailed model.

## Methods

### Network Formation

We consider a social network model, where the node degree is described by a Poisson distribution [50] with mean *ν*. In the simulation the network is first formed by a Poisson process: individuals acquire new neighbors with a constant probability per time step, and each neighbor connection has a constant probability per time step of being broken. This process is continued until the desired value of *ν *has been reached. The node degree distribution depends on the relative magnitude of *ϕ *and *φ*, the rate at which an individual forms a new neighbor and the rate at which an individual breaks up a neighbor relation, respectively. It can be shown that the average node degree in this process is 2*ϕ*/*φ*. The spread of disease is then studied through the resulting (now static) social network (with no demographic processes). Demographic processes are not included because the model represents a single epidemic moving through a small population on a timescale of months.

A number *I*_0 _of individuals out of a population with size *N *composed of susceptible individuals are selected at random and inoculated with the disease. There is a probability *β *per day at which an infectious node will transmit the disease to a neighboring susceptible node. Therefore, if a susceptible node has *n*_*inf *_infectious neighbors on a given day, the total probability *λ *that the node becomes infected on that day is(1)

The simulation time-step is one day, and each node's status is updated at the end of each day. This equation can be computed from basic probability theory.

### Decision Process

On any given day, a susceptible individual can choose either to vaccinate, or not to vaccinate (leaving open the possibility of vaccinating in future). If the payoff to vaccinate (*P*_*V*_) on a given day for a given individual is larger that the payoff not to vaccinate (*P*_*N*_), the individual will choose to vaccinate. Otherwise the individual will not vaccinate. Individuals weigh the benefits of vaccinating now (protection against the disease now, but with small vaccine risks and some probability the vaccine will not work) against the benefits of not vaccinating now (avoiding the risk of vaccinations and possibly avoiding infection as well, with the option to vaccinate in future if necessary, versus becoming infected at some point in future).

We assume that individuals become infectious when disease symptoms appear. We assume that individuals base vaccination decisions upon the presence of symptoms in a neighbor. This assumption is modeled according to the hypothesis that the attitudes are formed and modified as individuals acquire information about a process. When there is a lack of explicit communication, the only way of acquiring this information is through examination of individuals' neighbors [51]. Hence the *perceived *probability *λ*_*perc *_per day of being infected today, if an individual has *n*_*inf *_infected neighbors is given by(2)

where *β*_*perc *_is the perceived probability per day that the individual is infected by a single given infectious neighbor. In the following two subsections, we describe a Basic Model and an Extended Model with other assumptions about decision making.

### Basic Model

#### Vaccination and Natural History of Infection

We assume that the duration of latent period for an infected individual is drawn from a Gamma distribution with mean of 1/*σ *days and variance of *V*_*σ *_days^2^. The latent period is followed by an infectious period for a duration of time drawn from a Gamma distribution with mean of 1/*γ *and a variance of *V*_*γ *_days^2^. If exposed to a disease, the individual either dies with probability *d*_*inf *_due to fatal disease complications, or recovers gaining lifelong immunity with probability 1-*d*_*inf *_(we do not consider non-fatal outcomes such as long-term health conditions in this case). If an individual chooses to vaccinate, s/he will either vaccinate successfully with probability ϵ gaining lifelong immunity, or vaccinate unsuccessfully with probability 1-ϵ and remain susceptible to the disease. For a vaccinated individual, there is also *d*_*vac *_probability of death due to vaccine. In general, individuals have magnified perceptions of vaccine risks [52], so we assume a relatively high baseline perceived probability of death due to vaccine, *d*_*vac *_= 10^-3^. Baseline parameter values appear in Table 1.

**Table 1:** Baseline parameter values for SEIR-type infection.

Parameter	Meaning	Value	Reference
*N*	Population size	5000	assumption
*I*_0_	Initial number of individuals inoculated with smallpox	10	assumption
*ν*	Mean node degree	10	assumption, Ref. [64]
*τ*	Scaling constant for probability of infection from neighbours' neighbours	0.2	assumption
*β*	Probability of node-to-node transmission	0.02 day^-1^	Ref. [43]
*β*_*perc*_	Perceived probability of node-to-node transmission	0.02 day^-1^	Ref. [43]
1/*σ*	Mean duration of latent period	12 days	Ref. [65]
*V*_*σ*_	Variance of latent period	4 days^2^	Ref. [65]
1/*γ*	Mean duration of infectious period	19 days	Ref. [65]
*V*_*γ*_	Variance of infectious period (original model)	4 days^2^	Ref. [65]
*V*_*ι*_	Variance of infectious period (extended model)	4 days^2^	Ref. [65]
*V*_*κ*_	Variance of vaccine latent period	4 days^2^	Ref. [65]
*d*_*inf*_	Probability of death due to infection	0.3	Ref. [66]
*d*_*vac*_	Probability of death due to vaccine-related complications	0.001	assumption, Ref. [52]
ϵ	Vaccine efficacy	0.95	Ref. [43]
*α*	Payoff for individuals with continued susceptibility	40 life-years	Ref. [67]
*L*	Payoff for individuals with lifelong immunity	40 life-years	Ref. [67]

Parameter values selected are similar to those used in Ref. [49], and are intended to represent smallpox-type infections with high case fatality rates where transmission is dominated by close contact transmission to social contacts (e.g. household transmission, nosocomial transmission). Ref. [49] investigates the impact of variations in the node degree *ν*.

#### The Payoff Functions

**Figure 1:**
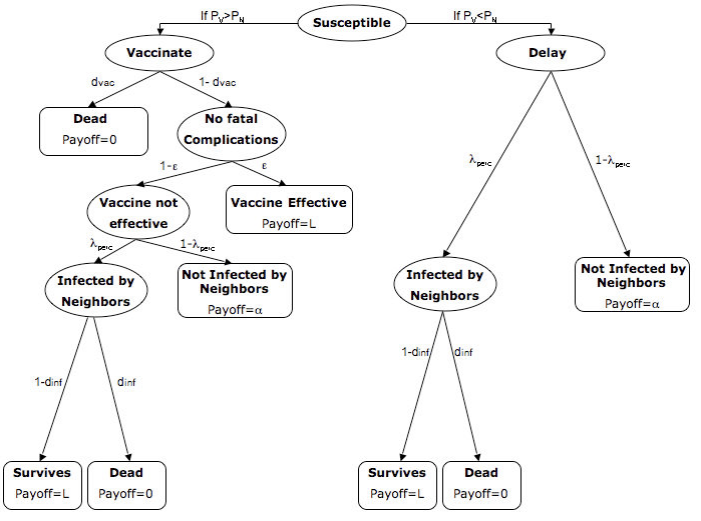
**Individual payoffs in the Basic Model**. Ellipses represent transition states while boxes represent formal states. Parameters on arrows denote transition probabilities and expressions in boxes denote payoffs for entering that state.

The payoff functions are expressed in terms of the number of life years *L *that the individual can expect to accrue as a result of their strategy choice. For an illustrative description of the decision process and different payoffs in the Basic Infection-Decision Model see Figure 1. Let us first consider the payoff *P*_*N *_for not vaccinating today. If the individual does not vaccinate, s/he is either infected today with perceived probability of infection *λ*_*perc *_or not with probability 1-*λ*_*perc*_. If the individual is infected today, s/he either dies due to fatal disease complications with probability *d*_*inf *_accruing no additional life-years, or recovers from the disease gaining lifelong immunity with probability 1-*d*_*inf *_and accruing *L *additional life years. Therefore, the payoff if the non-vaccinating individual is infected is (1 - *d*_*inf*_)*L*. Whereas if the non-vaccinating individual escapes infection today, then s/he receives a payoff of *α*, representing the individual's expected remaining life years under continued susceptibility (since s/he is not vaccinated nor infected today). Hence, the total payoff *P*_*N *_to an individual who does not vaccinate today is(3)

The parameter *α *is determined by the likelihood of being infected or of being compelled to vaccinate later on during the current epidemic outbreak in the population, and the likelihood that these result in death. Rather than expressing *α *in terms of weighted average of all future possibilities (which are numerous), we treat it as a constant parameter and select values which shed light on our research question. We expect *α *<*L*, since a person with immunity can expect to outlive a person without immunity, on average.

If we look at time horizons that are longer than the current epidemic, other factors may influence our assumptions about *α*. Namely, if vaccine-derived immunity wanes and the population is facing another outbreak in 20 years, then vaccination today may appear less beneficial, even if *α *<*L *still holds. In spite of that, these distant future events would be heavily discounted [53], hence we limit our time horizon just to the current outbreak. Although we expect *α *<*L*, it is not clear how much smaller it should be, therefore we will assume *α *= *L *(Ref. [49] explores the case *α *<*L*). We note that the baseline value of *L *does not change the model dynamics qualitatively or quantitatively, since *α *scales with *L *(subject to above assumptions about future outbreaks).

We next consider the payoff *P*_*V *_to vaccinate today. If the individual chooses to vaccinate, s/he will either vaccinate successfully with probability ϵ since the vaccine was efficacious, or unsuccessfully with probability 1 - ϵ if the vaccine was not efficacious. We make an assumption that there is a very small probability of dying from the vaccine, *d*_*vac*_. If the vaccine is efficacious but its outcome is death due to fatal complications from the vaccine then a person does not accrue any additional life years. On the other hand, if the vaccine is efficacious and the individual does not suffer any complications, then s/he receives a payoff(4)

Similarly, if the vaccine is not efficacious, then either the individual dies from fatal vaccine complications with probability *d*_*vac*_, accruing no additional life years, or the individual does not die (probability 1-*d*_*vac*_). In the latter case, the individual is either infected today with probability *λ*_*perc*_, or the individual escapes the infection today with probability 1-*λ*_*perc*_. If the individual is not infected today, s/he has escaped both death due to vaccine and infection and remains susceptible, so her/his payoff is *α*. On the other hand, if the individual is infected today, s/he suffers a probability *d*_*inf *_of dying due to the disease, accruing no additional life years. If s/he survives the infection (probability 1-*d*_*inf*_), her/his payoff is *L *accrued life years, or lifelong immunity. Hence, if the vaccine is not efficacious, the total payoff is(5)

and from Equations (4) and (5), we have(6)

On any given day, if(7)

then the individual decides to vaccinate. If the individual does not vaccinate today, they may still vaccinate in future according to the same decision rules, and with no memory of their previous decision history. If the individual vaccinates today, we assume they believe they are now protected and hence will not seek re-vaccination in future, which is a reasonable assumption given the high clinical efficacy of most vaccines when properly administered,

### Rationale for further analysis

Here we identify four simplifying assumptions of the Basic Model [49] that we relax in our current analysis. First, we look at a range of values for the probability of death due to infection and vaccination (rather than point values only) to see how they will influence infection dynamics.

Second, the previous model assumed no residual immunity to the disease. For relevance to the case where the population has experienced previous outbreaks it is desirable to investigate how and whether infection dynamics will change when a certain percentage of the population has residual immunity.

Third, the decision process in the previous model was binary: if the payoff to vaccinate exceeds the payoff not to vaccinate for an individual on a given day, s/he will always vaccinate and vice versa. Here, we assume that individuals vaccinate phenomenologically only with a certain probability when *P*_*V *_> *P*_*N*_. This can capture heterogeneity due to variation in health status risk perception etc.

Fourth, we previously assumed that individuals only vaccinate once symptoms appear in their neighbours. However, there may be cases where individuals vaccinate before the outbreak reaches that point, such as when their neighbors' neighbors become infected. This may be the case for infections where individuals can become infectious before exhibiting symptoms, for example. Moreover, we also assumed previously that the vaccine works immediately upon inoculation, which is a reasonable assumption in the case of smallpox since vaccination can prevent both disease and viral shedding in individuals who vaccinate within three days of becoming infected [54], but may not be a reasonable assumption for other infections. For instance, measles, mumps and rubella are diseases that are spread through close contact and it requires about 2 weeks for the influenza vaccine to provide protection against influenza virus infection [55] and about 20 days for developing MMR immunity [56]. Hence our assumption that the vaccine works right away is not always realistic. The effects of pre-emptive vaccination, and especially their interaction with assumptions about the latent period of infection and the time required for vaccines to mount an effective response to infection, could result in nontrivial dynamics. Therefore, we relaxed the simplifications of the original model by considering a range of possible disease latent periods, a range of possible time required for vaccination elicit full protection, and by including the infection status of neighbours' neighbours in the decision-making process of individuals.

Based on the preceding, our main research questions are the following: (i) What happens as perceived disease risk goes to zero (e.g. for a less dangerous disease like flu or chicken pox)-will voluntary ring vaccination still be effective? (ii) How do infection dynamics change when vaccine risk perception changes? (iii) How do infection dynamics change if a certain percentage of the individuals in the population have residual immunity to the disease? (iv) How do infection dynamics change if a probabilistic element is introduced in individual decision making? (v) How do the disease latent period, delay in time to effective vaccine-induced immune response, and pre-emptive vaccination based on infection status of neighbours' neighbours interact to determine infection-behaviour dynamics?

To answer the first three questions, we carried out further analyses of the Basic Model. To answer the fourth questions, we made a simple modification to the Basic Model. To answer the fifth question, we developed a substantially revised Extended Model as described in the following subsections.

### Extended Behaviour-Infection Model

#### Natural History of Infection

As before, the duration of the latent period for an infected individual is drawn from a Gamma distribution with mean of 1/*ι *and variance *V*_*ι *_days^2 ^and the duration of the latent period for a vaccinated individual (i.e., the duration of time before the vaccination is able to provide a protective response) is drawn from a Gamma distribution with mean of 1/*κ *and variance *V*_*κ *_days^2^.

#### The Payoff Functions

**Figure 2:**
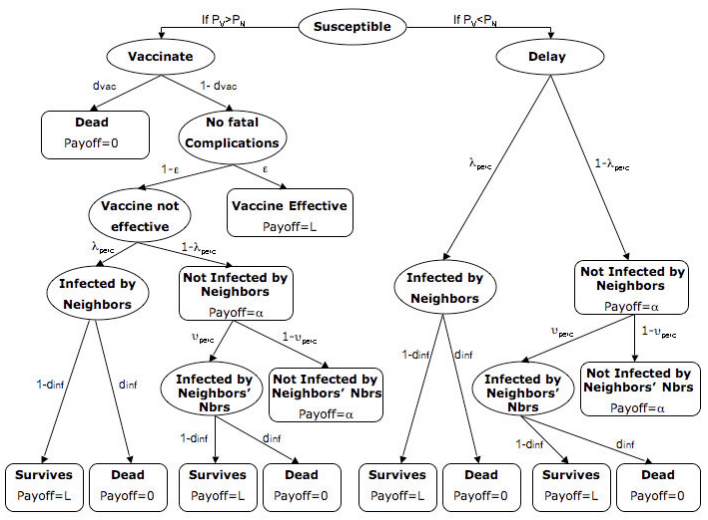
**Individual Payoffs in the Extended Model**. Ellipses represent transition states while boxes represent formal states. Parameters on arrows denote transition probabilities and expressions in boxes denote payoffs for entering that state.

Here we modify the previous payoff functions so that individuals take into account the number of infectious neighbors' neighbors when deciding whether to vaccinate. For an illustrative description of the decision process and different payoffs in the Extended Model see Figure 2. Let us first consider the payoff *P*_*N *_for not vaccinating today. As before, if the individual does not vaccinate, s/he is either infected today with perceived probability of infection *λ*_*perc *_or not with probability 1-*λ*_*perc*_. If the individual is infected today, s/he either dies due to fatal disease complications with probability *d*_*inf *_accruing no additional life years, or recovers from the disease gaining lifelong immunity with probability 1-*d*_*inf *_and accruing *L *additional life years.

On the other hand, if the individual is not infected today by her/his immediate neighbors, then s/he is either infected in the near future by her/his infectious neighbors' neighbors or not. If the individual is infected by her/his infectious neighbors' neighbors, s/he either dies due to fatal disease complications with probability *d*_*inf *_accruing no additional life years, or recovers from the disease gaining lifelong immunity with probability 1-*d*_*inf *_and accruing *L *additional life years. If the non-vaccinating individual is not infected by her/his infectious neighbors' neighbors, s/he receives a payoff of *α *(same value and the meaning as in our original model).

We assume that the *perceived *probability *ϑ*_*perc *_per day of being infected by infectious neighbors' neighbors is given by(8)

where *m*_*inf *_represents the number of infectious neighbors' neighbors of an individual and *τ *is a scaling constant that controls how quickly individuals get infected by their infectious neighbors' neighbors-the individuals with more infectious neighbors' neighbors have a higher probability of getting infected than the individuals with less infectious neighbors' neighbors. (We note that this equation makes the simplifying assumption that individuals have information on which of their neighbours' neighbours are infected, but not which of their neighbours' neighbours have been vaccinated, and this could potentially influence decision-making.) Hence, the total payoff *P*_*N *_to an individual who does not vaccinate today is(9)

We next compute the payoff *P*_*V *_to vaccinate today. If the individual chooses to vaccinate, s/he will either vaccinate successfully with probability ϵ since the vaccine was efficacious, or unsuccessfully with probability 1 ϵ if the vaccine was not efficacious. We make an assumption that there is a very small probability of dying from the vaccine, *d*_*vac*_. If the vaccine is efficacious but its outcome is death due to fatal complications from the vaccine then a person does not accrue any additional life years. On the other hand, if the vaccine is efficacious and the individual does not suffer any complications, then s/he receives a payoff(10)

Similarly, if the vaccine is not efficacious, then either the individual dies from fatal vaccine complications with probability *d*_*vac*_, accruing no additional life years, or the individual does not die (probability 1-*d*_*vac*_). In the latter case, the individual is either infected today with probability *λ*_*perc*_, or the individual escapes the infection today with probability 1-*λ*_*perc*_.

If the individual is not infected today by her/his immediate neighbors, then s/he is either infected in the near future by her/his infectious neighbors' neighbors or not. If the individual is infected by her/his infectious neighbors' neighbors, s/he either dies due to fatal disease complications with probability *d*_*inf *_accruing no additional life years, or recovers from the disease gaining lifelong immunity with probability 1-*d*_*inf *_and accruing *L *additional life years. If the non-vaccinating individual is not infected by her/his infectious neighbors' neighbors, s/he has escaped both death due to the vaccine and infection and remains susceptible, so s/hereceives a payoff of *α*.

On the other hand, if the individual is infected today, s/he suffers a probability *d*_*inf *_of dying due to the disease, accruing no additional life years. If s/he survives the infection (probability 1-*d*_*inf*_), her/his payoff is *L *accrued life years, or lifelong immunity. Hence, if the vaccine is not efficacious, the total payoff is(11)

and from equations (10) and (11), we have(12)

And again, on any given day, if

then the individual decides to vaccinate. Otherwise they may still vaccinate in future according to the same decision rules, and with no memory of their previous decision history.

## Results and Discussion

### Basic Model

#### Disease and Vaccine Risk

**Figure 3:**
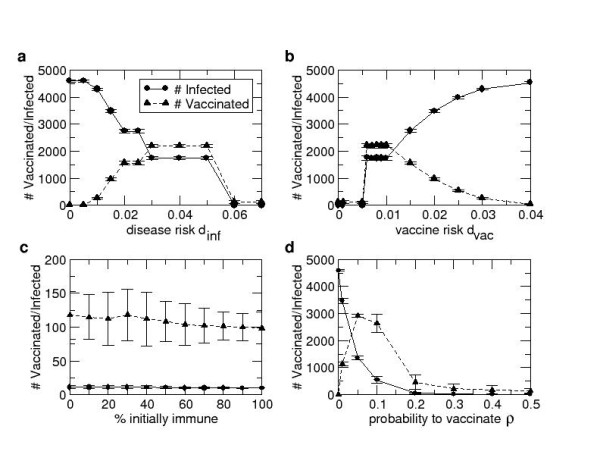
**Results from the Basic Model**. Dependence of final epidemic size and final number vaccinated on: probability of death due to disease *d*_*inf *_when *d*_*vac *_= 0.001 (**a**), probability of death due to vaccine *d*_*vac *_when *d*_*inf *_= 0.03 (**b**), percentage of population with previous immunity (**c**), and probability to vaccinate *ρ *(**d**) in the Basic Model. Error bars represent two standard deviations from the mean across 20 simulations per data point. Note that *d*_*inf *_= 0.3 (Table 1) lies to the right of the range illustrated in Figure 1a; we did not plot the results for *d*_*inf *_> 0.07 because they are qualitatively unchanged from the case *d*_*inf *_= 0.07.

When varying the probability of death due to disease *d*_*inf*_, and the probability of death due to vaccine *d*_*vac*_, three types of dynamics are observed (Figure 3a,b). First, for *d*_*inf *_≥ 0.06 and *d*_*vac *_≤ 0.005, a negligible final size of the epidemic and a very small number vaccinated occurs. In this case, all individuals with at least one infectious neighbor vaccinate immediately, preventing disease transmission. Secondary and tertiary transmissions are limited and rare and occur only because of vaccine failures in some individuals. Therefore, the epidemic is successfully controlled through voluntary ring vaccination.

Second, when *d*_*inf *_≤ 0.005 or *d*_*vac *_≥ 0.06 (or when *r *≤ 0.2 [see Additional File 1]) individuals stop vaccinating, resulting in large final size since there are no control measures to prevent the infection from spreading. A third outcome between those thresholds, when 0.005 <*d*_*inf *_< 0.06 and 0.005 <*d*_*vac *_< 0.06, is a large number vaccinated and a large final size since individuals with more infectious neighbors perceive disease more risky than the vaccine and will normally vaccinate, while individuals with less infectious neighbors perceive vaccine more risky than the disease and do not always vaccinate, allowing the disease to percolate through the population.

In Figure 3b, it is clear that there are parameter regimes where *d*_*vac *_≈ 0.03 is much lower than *d*_*inf *_= 0.3, but nonetheless individuals are predicted not to vaccinate and infection is predicted to spread throughout the network. This appears counterintuitive, however, it stems from the fact that the payoff not to vaccinate is a function of both the probability of death and the probability of being infected. At the parameter values of Table 1, the per-day probability of transmission from a susceptible to an infected person is 0.02, which is relatively low. By comparison, choosing to vaccinate today means the individuals takes an instantaneous risk. Therefore, even if *d*_*vac *_is less than *d*_*inf*_, individuals may choose not to vaccinate, preferring to take their chance that their social contacts will soon recover and hence no longer post an infection risk to them. A simple calculation based on these simulation results [see Additional File 1] shows that in order for an outbreak to be prevented, under our baseline parameter assumptions (Table 1), the following condition needs to be satisfied:(13)

or equivalently(14)

where *r *stands for perceived relative risk (*r *= *d*_*vac*_/*d*_*inf*_). It is possible to arrive at a similar conclusion by analyzing the equation:(15)

which was derived in Ref. [49] from equations (3) and (6), and represents a condition under which an individual will vaccinate if s/he has at least one infectious neighbor in the Basic Model. Under our baseline parameters (Table 1), it can easily be shown that the equation (15) holds true whenever(16)

which is consistent with Equation (14). Hence, individuals need to perceive the vaccine to be approximately 50 times safer than the disease before they will chose to vaccinate.

#### Pre-existing Immunity

Here we study the case where a certain proportion of the population has immunity from previous outbreaks. We assume that individuals are not aware of their pre-existing immunity and will follow the same decision process as before. However, if exposed to the disease, the individuals with pre-existing immunity will not get infected and hence will not transmit the disease. We note that in populations where pre-existing immunity from previous outbreaks is based on previous exposure to natural infection only (and not vaccination), individuals with pre-existing immunity are more likely to be aware of this fact due to the highly characteristic symptoms of smallpox infection; therefore this assumption may not be valid for those populations without previous access to vaccine. However, in cases where ring vaccination was widely applied in previous outbreaks in the population, individuals may not know their immune status due to the possibility of waning vaccine-derived immunity and hence may conservatively assume they no longer have immunity.

We observe that the final size and number vaccinated are not strongly dependent on percentage of the population with pre-existing immunity (Figure 3c). This is exactly what we expected to see: individuals vaccinate as soon as they find out that they have an infectious neighbor, preventing the transmission of the infection, regardless of whether they have previous immunity or not. However, we see slightly more secondary transmission when the percent immune is low.

The current social contact network is not age structured, even though social contacts tend to be age structured. Therefore there was no mechanism in our model to have individuals with residual immunity preferentially make contact with other individuals that have residual immunity, but age structuring of the population might well cause such a preference. Hence, the lack of dependence of final size and number vaccinated on percentage of the population with pre-existing immunity may, in part, result from their being well mixed in the population.

#### Probabilistic Decision Process

To study the effect of probabilistic decision-making on disease dynamics we varied the probability (*ρ*) with which an individual vaccinates when *P*_*V *_> *P*_*N*_. In this case the decision to vaccinate or not is slightly different than in the original model and is based upon the following: if

then the individual does not vaccinate today (but may still vaccinate in the future), but if

then the individual vaccinates with a certain probability *ρ*. For this scenario, we again observe three possible types of dynamics. When varying *ρ*, we observe that the final size increases dramatically as *ρ *falls below 0.2 (Figure 3d). The number vaccinated is highest for 0.05 ≤ *ρ *≤ 0.2. For *ρ *< 0.05, the number vaccinated is small since *ρ *is small and therefore very few individuals with infectious neighbors are allowed to vaccinate and as a result, the infection easily spreads to over 90% of the population. For *ρ *> 0.2 the number vaccinated as well as the final size are low because the outbreak is quickly contained through voluntary ring vaccination.

Even though our network model is not dynamic, these results are qualitatively comparable to the results of a lattice gas cellular automaton model of Ref. [57] which shows that the severity of an epidemic also increases as the proportion of vaccinated neighbors of an infectious individual decreases. In conclusion, as long as a certain proportion of the population acts rationally and vaccinates in the presence of infectious neighbors on any given day, the outbreak will be effectively controlled through voluntary ring vaccination. This proportion could be as low as 5% (*ρ *= 0.05), at our baseline parameter values!

#### Extended Model

Table 2 defines parameter values used in the Extended Model. Tables 3 and 4 contain the results from the Extended Model, where we consider the final size of the epidemic and final number vaccinated as a function of the duration of the disease latent period (1/*ι*) and the duration of the vaccine latent period (1/*κ*). We observe 2 regions with relatively low numbers of vaccinated and infectious individuals: (i) when 1/*κ *≤ 15 days and (ii) a band slightly off the main diagonal when 1/*ι *is marginally less than 1/*κ*. Otherwise, we observe large final size and number vaccinated.

**Table 2:** Parameter definitions for the Extended Model.

Parameter	Meaning
*α*	Payoff to a person who remains susceptible today
*L*	Payoff to a person who has acquired immunity, either through vaccine or through infection and who did not experience long-term complications from vaccine or infection
*n*_*inf*_	Number of infectious neighbors for an individual on a given day
*m*_*inf*_	Number of infectious neighbors' neighbors for an individual on a given day
*λ*	Probability per day of an individual becoming infected by infectious neighbors
*λ*_*perc*_	Perceived probability per day of an individual becoming infected by infectious neighbors
*ϑ*_*perc*_	Perceived probability per day of an individual becoming infected by infectious neighbors' neighbors
*ρ*	Probability with which an individual will vaccinate today if her/his *P*_*V *_> *P*_*N *_today
1/*ι*	Duration of the latent period for an infectious individual in the extended model
1/*κ*	Duration of the latent period for a vaccinated individual in the extended model

**Table 3:** The Extended Model results.

Number of Days	0	10	20	30	40	50
0	**12.1** *1226.56*	**75.55** *3774.8*	**594.25** *4924*	**1782** *4955.55*	**3254.6** *4959*	**4118.9** *4957.9*

10	**11.85** *116.9*	**36.05** *2766.3*	**100.30** *4039.55*	**405.8** *4890*	**1000.5** *4948.5*	**1633.3** *4954.8*

20	**12.9** *132.15*	**796.45** *3896.75*	**46.8** *3166.8*	**94.95** *3946.9*	**344.8** *4864.4*	**725.95** *4939*

30	**11.75** *120.6*	**745.1** *3685.1*	**1674.8** *4683.35*	**49.95** *3231.2*	**105.9** *4202.05*	**343.45** *4870.9*

40	**12.5** *124.45*	**803.25** *3918.5*	**1645.55** *4666.7*	**1891.55** *4754.8*	**44.15** *2952.6*	**105.15** *4250.85*

50	**12** *120.1*	**780.3** *3870.35*	**1560.45** *4439.1*	**1825.4** *4735.25*	**1930.7** *4762.65*	**47.35** *3132.9*

Rows represent the duration of the disease latent period 1/*ι*, while columns represent the duration of the vaccine latent period 1/*κ*. Final epidemic size is shown using bold numbers and final number vaccinated is italicized. See Table 4 for the corresponding standard deviation values.

**Table 4:** Standard deviation values corresponding to the results in Table 3.

Number of Days	0	10	20	30	40	50
0	± **1.37** ± *219.21*	± **21.29** ± *384.38*	± **111.24** ± *17.67*	± **218.91** ± *5.69*	± **203.81** ± *2.65*	± **72.88** ± *4.07*

10	± **1.39** ± *18.75*	± **4.14** ± *234.98*	± **27.99** ± *942.46*	± **67.78** ± *24.52*	± **106.24** ± *4.67*	± **121.63** ± *4.62*

20	± **1.73** ± *28.30*	± **43.25** ± *80.46*	± **9.18** ± *302.86*	± **31.94** ± *948.69*	± **55.93** ± *29.19*	± **66.73** ± *5.96*

30	± **1.64** ± *24.81*	± **180.08** ± *854.25*	± **59.46** ± *34.15*	± **9.12** ± *331.18*	± **24.78** ± *286.69*	± **49.57** ± *28.25*

40	± **1.80** ± *26.15*	± **47.22** ± *90.17*	± **43.12** ± *21.34*	± **37.54** ± *19.57*	± **12.43** ± *732.02*	± **20.16** ± *214.17*

50	± **1.67** ± *24.34*	± **43.25** ± *80.63*	± **359.64** ± *1018.59*	± **68.03** ± *28.17*	± **47.97** ± *18.96*	± **9.51** ± *390.62*

Rows represent the duration of the disease latent period 1/*ι*, while columns represent the duration of the vaccine latent period 1/*κ*. Standard deviation values for the final epidemic size (bold numbers) and for the final number vaccinated (italicized numbers) are calculated based on the results from 20 simulations per data point.

First of all we consider the region where 1/*κ *≤ 15 days. We see small final size and small number vaccinated when 1/*κ *≤ 10 days since knowing that it takes some time for the vaccine to work, individuals vaccinate right away if they have infectious neighbor(s) hence the epidemic is relatively quickly contained through voluntary ring vaccination. For 10 days < 1/*κ *< 15 days, it takes longer for the vaccine to work therefore even if the individuals with infectious neighbor(s)/neighbors' neighbor(s) vaccinate right away, they may not be able to prevent the infection. This results in a significant increase in the number of vaccinated individuals and the final size in this region. Similarly as 1/*ι *approaches 50 days for a given value of 1/*κ*, individuals with infectious neighbor(s)/neighbors' neighbor(s) start waiting more and more before they choose to vaccinate, overestimating how much time they and in those cases not being able to prevent the infection.

Secondly, we consider the area about the main diagonal. The epidemic is very quickly controlled when the two latent periods are exactly the same, since individuals with infectious neighbor(s), or infectious neighbors' neighbor(s), will vaccinate right away, understanding that it takes the same amount of time for the vaccine to work as it takes for them to become infected. Hence we see small final size and small number vaccinated on the main diagonal. Above it, we see a wide band of slightly higher values. In this case 1/*κ *> 1/*ι *and being aware that it takes longer for the vaccine to work than to get infected from his/her immediate neighbor, individuals vaccinate as soon as their neighbors' neighbor becomes infectious and therefore the epidemic is effectively controlled. On the other hand, below the main diagonal where 1/*κ *< 1/*ι*, we see a narrow band of significantly higher values than on the main diagonal. In this case some individuals overestimate how much time they have to vaccinate and are not able to prevent infection, which results in a large final size and number vaccinated.

Above and below the band of relatively low values around the main diagonal, we see very large final size and number vaccinated. Below the band where 1/*κ *≪ 1/*ι*, this is the case since the individuals with infectious neighbor(s)/infectious neighbors' neighbor(s) do not think to vaccinate right away since 1/*ι *is now large. Once they do vaccinate, it takes 15–45 days for the vaccine to work so most of the vaccinated individuals end up becoming infected. Alternatively, above the band where 1/*κ *≫ 1/*ι*, we see high numbers of vaccinated and infectious individuals since the vaccine does not work fast enough even if the individuals vaccinate as soon as they have infectious neighbor(s)/infectious neighbors' neighbor and most of the population ends up becoming infected.

Although the results may differ for other assumptions and parameter values relating to the decision-making process, these results show how disease latent period, vaccine latent period and decision-making can interact to produce a wide range of results at the population level.

It can be argued that the network we analyze is technically not a static network, because dead individuals would normally be removed from a social contact network and hence the network links change through the course of the outbreak. However, the network is static in the important sense that no links through which transmission of disease is possible are formed or broken up during the simulation: individuals who leave the infectious compartment are either recovered and immune for life, or dead, but in either case they will not transmit any more infection during the current outbreak. Therefore the dynamics will be the same regardless of whether dead individuals are removed from the network in the course of the simulation (and assuming that dead individuals are not replaced with susceptible individuals, which is a valid assumption for the course of a single outbreak). We note that the case fatality rate is still relevant to the dynamics through the behaviour function, however, since a more dangerous disease will change the individual willingness to vaccinate.

Here we make the idealized assumption that individuals will vaccinate on any given day if *P*_*V *_> *P*_*N *_and, should they choose not to vaccinate, will not have a memory of that decision in the future. We emphasize that this is a highly simplified description of individual decision-making that may not hold in real populations. For instance, the role of omission bias in pertussis vaccination has been previously explored [58], prospect theory may have some validity in individual vaccine decision-making processes [59], and bounded rationality may be important in developing assumptions about individual decision-making as well [17]. Finally, we also emphasize our assumption that individuals are not influenced by-and do not have information regarding-the global status of the epidemic, such as the presence of infection in other parts of the network. However, in reality, some individuals may choose to pre-emptively vaccinate despite not having any infected social contacts. This factor and similar factors such as the effects of media coverage could be included in future models.

## Conclusion

Numerous studies have shown that human behavior has a notable effect on infection dynamics and that its incorporation into epidemic models is essential for making more accurate predictions about disease spread, evaluating different control measures and developing appropriate policies. The previous literature on behavior-infection models of disease transmission has usually focused on homogeneously mixing populations without any geographical or social contact structure [5], even though heterogeneity in network structure significantly affects transmission dynamics [26,29,30,33-35]. Few models have used network or individual-based approaches. Our earlier work [49] revealed important differences in behavior-infection dynamics between contact-structured and contact-unstructured populations, for instance the incentive for an individual to vaccinate under a voluntary vaccination policy. It also demonstrated that spatial localization of transmission can enable eradication of a disease under a voluntary vaccination policy, which contradicts the conclusions of homogeneously mixing models which normally predict that eradication is impossible under such a policy. This result confirms previous findings about the importance of population structure and the strong effect it has on the disease transmission [29,30,34,35,60]. It also suggests that incorporating all scales, from individuals and smaller communities to large cities with distinct structures and transmission probabilities, into the model may be important [45]. Even though this represents a considerable challenge for the researchers, since it requires knowledge about how transmission varies with social or geographical space, it should be explored in greater depth.

However, Ref. [49] made simplifying assumptions concerning disease natural history, pre-exisitng immunity and human behavior. Here, we relaxed those assumptions and showed that the qualitative outcome of rational, self-interested behaviour under a voluntary vaccination policy can vary significantly depending on interactions between contact structure, perceived vaccine and disease risks, and the way that individual vaccination decision-making is modeled and that disease transmission dynamics is not strongly dependent on percentage of population with pre-existing immunity. In general, based on our results we distinguish three qualitatively different cases regarding the possible outcomes of the epidemic in terms of the final size and total number vaccinated depending on risk and behavior variables: (1) small total number vaccinated and final epidemic size, due to rapid control through voluntary ring vaccination, (2) large total number vaccinated and significant final epidemic size, due to imperfect voluntary ring vaccination, and (3) little or no vaccination and large final epidemic size, corresponding to little or no voluntary ring vaccination. These simulations indicated that alignment of socially and individually optimal results (outcome (1) above), which generally does not occur in homogeneous mixing models, occurs for a fairly broad range of parameter values investigated here. Finally, our model illustrates the sensitivity of epidemiological systems by showing that slight changes in values of the parameters such as perceived disease or vaccine related risk or vaccination behaviour-related parameters, can lead to a significant change in population-level outcomes such as final size and total number vaccinated.

Ref. [14] showed that the expected vaccine uptake is less than the eradication threshold for any perceived relative risk *r *> 0, formalizing the argument that it is impossible to stop an outbreak through voluntary vaccination when individuals act according to their own interest [8-13,15-18,61]. Our results indicate that it is possible to eradicate a disease through voluntary vaccination when individuals act according to their own interest even when *r *> 0, confirming the result from our earlier work [49]. More specifically, we showed that the outbreak is controlled when 0.02 <*r *< 0.2 and completely prevented when *r *≤ 0.02 on average.

Although we have relaxed many of the simplifying assumptions made in the previous paper [49], some still remain. For instance, we assumed that *α *= *L*. For the case *α *<*L*, we would expect to see higher total number vaccinated and as a result, lower final epidemic size, on average. In addition, we used a static, random network to investigate the issues in the current study. Considering the findings from earlier analysis that show that the spread of disease depends on the network structure [29,30,34,35,60], the predictions may be very different for other network types. Therefore, the need for incorporating other types of network structures, including those that accurately represent real networks in explicit geographical regions [39,40] as well as adaptive networks [62], into epidemic models is obvious and is something that should be further investigated.

In order to refine their understanding of epidemiology of infectious diseases, researchers have been introducing greater biological realism into models [5,7,63-67]. Here, by relaxing the simplifying assumptions made in the previous model by the authors [49], we have been able to better understand what changes and what stays the same under alternative assumptions about individual decision-making and infectious disease epidemiology.

Contact network models mathematically formalize contact patterns between the individuals that govern disease transmission and hence produce more accurate results for predicting disease spread through heterogeneous host populations than the models with unstructured populations, especially for disease that are spread through close contact such as sexually transmitted infections [36]. Also, incorporating human behavior into epidemic models has been proved to be essential, since it determines infection dynamics [5]. Our results show that interactions between human behavior and the role of close contacts in disease transmission may be an important factor for determining the feasibility of outbreak control under voluntary vaccination policies. Hence, using network-based behavior prevalence models is crucial for obtaining more accurate predictions about disease spread, evaluating different control measures and developing appropriate vaccination policies.

## Competing interests

The authors declare that they have no competing interests.

## Authors' contributions

AP wrote the simulation code, helped design the model, performed simulation experiments, and wrote first drafts of the manuscript. CTB conceived of the project, designed the model, and finalized the manuscript.

## Pre-publication history

The pre-publication history for this paper can be accessed here:

http://www.biomedcentral.com/1471-2334/9/77/prepub

## Supplementary Material

Additional file 1 **Additional Derivations**. Additional File 1 describes derivation of Equation (13) and computation of the relative risk. Click here for file
