# Supplementary material for: A simulation analysis to characterize the dynamics of vaccinating behaviour on contact networks
Source: BMC Infect Dis. 2009 May 28;9:77. doi: 10.1186/1471-2334-9-77 (PMC2695470; doi:10.1186/1471-2334-9-77)
Supplement: Additional file 1 — Additional Derivations. Additional File 1 describes derivation of Equation (13) and computation of the relative risk. [file 1471-2334-9-77-S1.pdf]

Additional File 1: Additional Derivations

Derivation of Equation (13)

By looking at the results from varying the probability of death due to disease d_{inf}, we see that an outbreak is prevented as long as $d_{inf} \geq 0.06$. Considering that $d_{vac} = 0.001$ in all of the simulations, we can say that epidemic will be prevented as long as

$$d_{inf} \geq 60 d_{vac} \tag{17}$$

Similarly, by looking at the results from varying probability of death due to vaccine d_{vac}, we see that infection does not spread for $d_{vac} \leq 0.005$. Since $d_{inf} = 0.3$ in all of the simulations, we can say that disease transmission will be prevented as long as $d_{vac} \leq 1/60\ d_{inf}$ or $d_{inf} \geq 60 d_{vac}$, which is the same as Equations (13) and (17).

Relative Risk Calculation

By looking at the results from varying the probability of death due to disease d_{inf}, we see that individuals stop vaccinating when $d_{inf} \leq 0.005$. Considering that $d_{vac} = 0.001$ in all of the simulations, the relative risk becomes:

$$r = \frac{0.001}{\leq 0.005} \Rightarrow r \geq 0.2 \tag{18}$$

Similarly, by looking at the results from varying the probability of death due to vaccine d_{vac}, we see that individuals stop vaccinating when $d_{vac} \geq 0.06$. Considering that $d_{inf} = 0.3$ in all of the simulations, the relative risk becomes:

$$r = \frac{\geq 0.06}{0.3} \Rightarrow r \geq 0.2 \tag{19}$$
